# Supplementary material for: Phage Genome Diversity in a Biogas-Producing Microbiome Analyzed by Illumina and Nanopore GridION Sequencing
Source: Microorganisms. 2022 Feb 4;10(2):368. doi: 10.3390/microorganisms10020368 (PMC8879888; doi:10.3390/microorganisms10020368)
Supplement: Supplementary file 1 [file microorganisms-10-00368-s001.zip › microorganisms-1543547-supplemenraty figures.pdf]

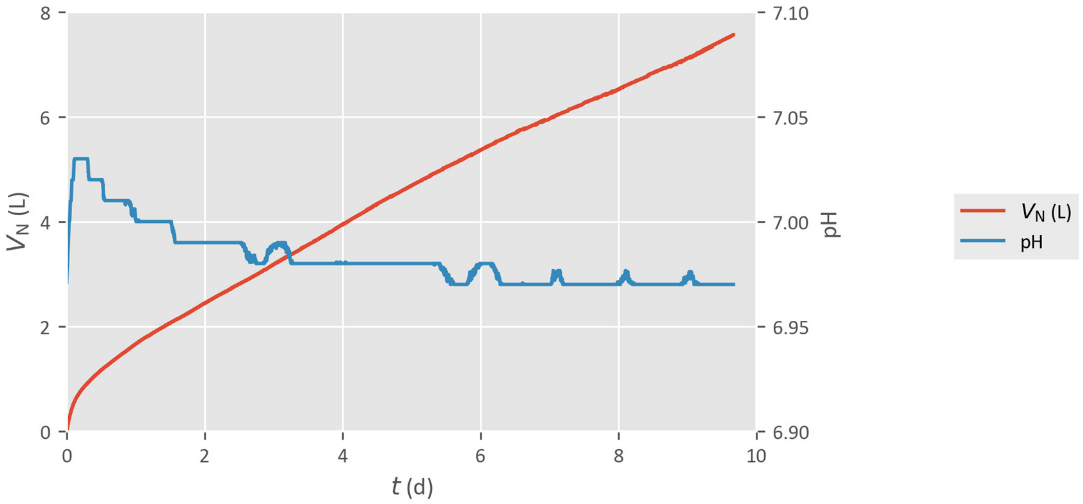

(**A**)

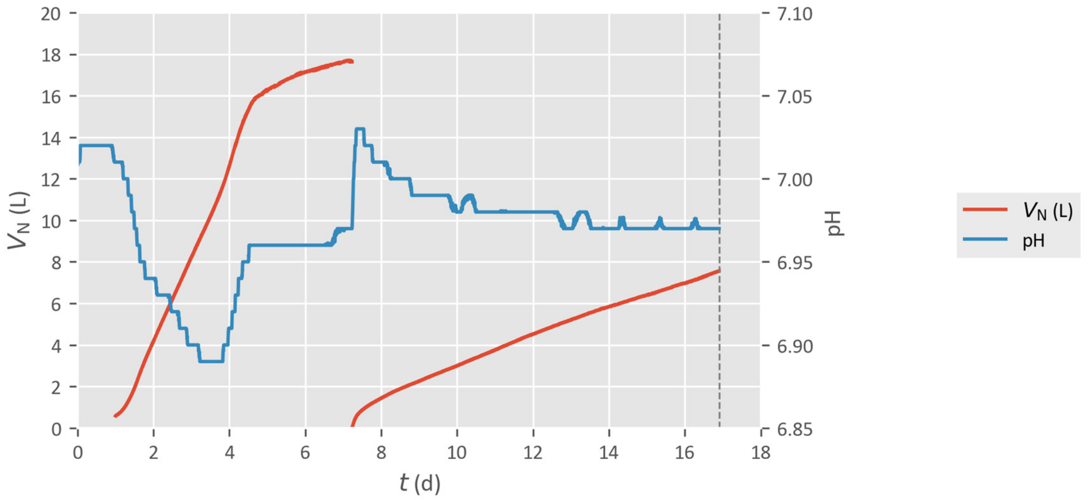

(**B**)

Figure S1. (**A**) Biogas formation VN (L) and pH (moving average, window size = 15) during the anaerobic digestion of carboxymethyl cellulose (CMC) in the investigated continuous stirred tank reactor (CSTR) prior to sampling at t = 9.7 d. CMC was added to a final concentration of 0.5% at t = 0 (YB, CMC = 92 mL g_{ODM}^{-1}). (**B**) Biogas formation VN (L) and pH (moving average, window size = 15) during the anaerobic digestion of microcrystalline cellulose (MCC) and carboxymethyl cellulose (CMC) in the investigated continuous stirred tank reactor (CSTR). Addition of 24 g MCC at t = 0 d (YB, MCC = 735 mL gODM-1), addition of 82.5 g CMC at t = 7.25 d (YB, CMC = 92 mL g_{ODM}^{-1}), and sampling at t = 16.9 d (depicted by a vertical dashed line).

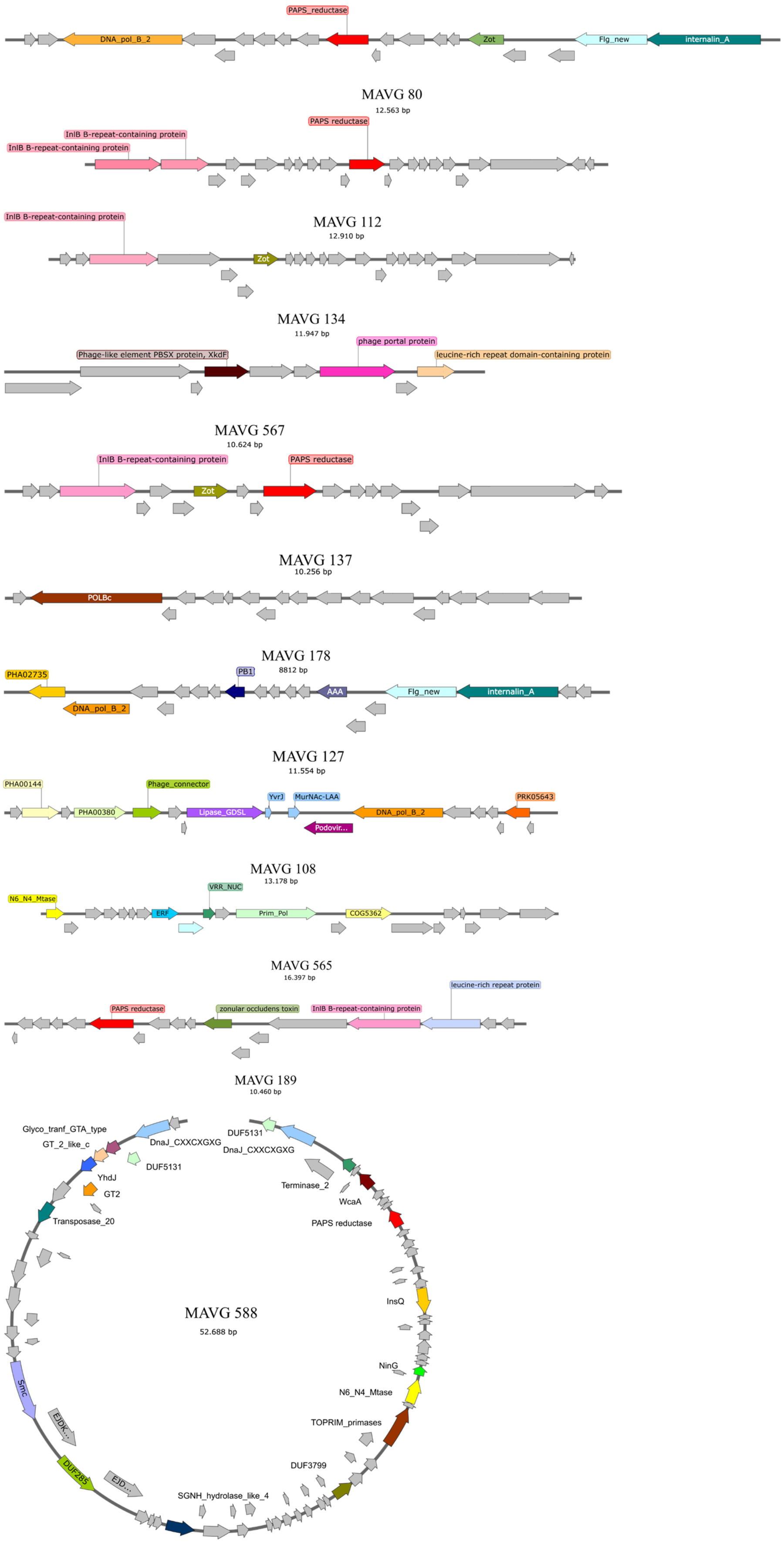

Figure S2. Linear and circular representations of manually annotated genomes of MAVGs 80, 112, 134, 567, 137, 178, 127, 108, 565, and 189. ORFs that were found in two or more genomes are colored with the same color (e.g., PAPS

reductase in bright red). Hypothetical proteins are depicted in grey and are not labeled. The ORFs identified are indicated by arrows in the direction in which they are translated. Genomes were generated with Snapgene.
